# Acute ischemic stroke in childhood: a comprehensive review

**DOI:** 10.1007/s00431-021-04212-x

**Published:** 2021-07-29

**Authors:** Mario Mastrangelo, Laura Giordo, Giacomina Ricciardi, Manuela De Michele, Danilo Toni, Vincenzo Leuzzi

**Affiliations:** 1 Child Neurology and Infantile Psychiatry Unit, Department of Human Neuroscience, Sapienza University of Rome, Rome, Italy; 2 Emergency Department Stroke Unit, Department of Human Neurosciences, Sapienza University of Rome, Rome, Italy

**Keywords:** Stroke, Neuroimaging, Diagnostic protocol, Thrombolysis, Recanalization therapies, Children

## Abstract

This review provides an updated analysis of the main aspects involving the diagnosis and the management of children with acute ischemic stroke. Acute ischemic stroke is an emergency of rare occurrence in children (rate of incidence of 1/3500 live birth in newborns and 1–2/100,000 per year during childhood with peaks of incidence during the perinatal period, under the age of 5 and in adolescence). The management of ischemic stroke in the paediatric age is often challenging because of pleomorphic age-dependent risk factors and aetiologies, high frequency of subtle or atypical clinical presentation, and lacking evidence-based data about acute recanalization therapies. Each pediatric tertiary centre should activate adequate institutional protocols for the optimization of diagnostic work-up and treatments.

*Conclusion*: The implementation of institutional standard operating procedures, summarizing the steps for the selection of candidate for neuroimaging among the ones presenting with acute neurological symptoms, may contribute to shorten the times for thrombolysis and/or endovascular treatments and to improve the long-term outcome.

**Table:** 

**What is Known:** *•Acute ischemic stroke has a higher incidence in newborns than in older children (1/3500 live birth versus 1–2/100,000 per year).* *•Randomized clinical trial assessing safety and efficacy of thrombolysis and/or endovascular treatment were never performed in children*	
**What is New:** *•Recent studies evidenced a low risk (2.1% of the cases) of intracranial haemorrhages in children treated with thrombolysis.* *•A faster access to neuroimaging and hyper-acute therapies was associated with the implementation of institutional protocols for the emergency management of pediatric stroke.*

**What is Known:**

*•Acute ischemic stroke has a higher incidence in newborns than in older children (1/3500 live birth versus 1–2/100,000 per year).*

*•Randomized clinical trial assessing safety and efficacy of thrombolysis and/or endovascular treatment were never performed in children*

**What is New:**

*•Recent studies evidenced a low risk (2.1% of the cases) of intracranial haemorrhages in children treated with thrombolysis.*

*•A faster access to neuroimaging and hyper-acute therapies was associated with the implementation of institutional protocols for the emergency management of pediatric stroke.*

## Introduction

According to the American Heart Association- American Stroke Association definition, ischemic stroke is an episode of neurological dysfunction caused by focal cerebral, spinal, or retinal infarction involving a specific vascular district, and presenting with symptoms lasting for more than 24 h or until death and neuroimaging, pathological, and/or other objective evidences of focal ischemic injuries [1].

The occurrence of ischemic stroke in pediatric age, despite its rarity, implicates age-dependent peculiarities in terms of risk-factors, etiopathogenesis, clinical presentations, and therapeutic approaches [2–10]. The lack of validated evidence-based data about thrombolytic and endovascular treatments in children represents the main limit towards the prevention of life-threatening or disabling sequelae [2].

The aim of this review is to provide an updated overview on the current evidences and an insight into the future perspective on the management of acute ischemic stroke in childhood. A detailed analysis of epidemiological, clinical presentation, and management of neonatal stroke was beyond the scope of the authors even if the readers may find useful updates in the provided references [7, 11–13]

## Epidemiology

The interpretation of data on incidence and risk factors of acute ischemic stroke in children is complicated by several differences in methods (e.g., identification of the cases via diagnostic code searches, variable age-ranges of the studied cohorts, large predominance of retrospective studies on selected cohorts of patients) [2–8]. The reported incidence is higher in newborns than in older children (1/3500 live birth versus 1–2/100,000 per year) with a ratio of approximately six times higher [2]. The peaks of incidence were evidenced in the perinatal period (5–13/100,000 live births), in children under the age of 5 (0.38/100,000 per year) and in adolescence (0.48–0.6/100,000 per year) [2–8]. Two recent prospective studies, in Canada and Germany, reported lower overall incidence ranges (0.41/100,000–1.72/100,000 children/year) even if the German study did not include newborns [4, 5].

A higher predisposition to an earlier onset was highlighted in Asian and black children, because of a higher incidence of concurrent chronic disorders (with a relative risk of 2.14 and 2.28, respectively, for Asian and black children, compared to 1.34 of other ethnicities) [8]. Data about the relationships between age of clinical presentation and sexes are discordant [9, 10]. Two-old retrospective studies reported a slightly increased risk in males, even after stratification by age and aetiologies (relative risk of 1.25), while a more recent prospective population-based study performed in the South of England did not detect any statistically significant differences [8–10].

## Etiopathogenesis and risk factors

Pediatric ischemic stroke may be caused by several factors inducing thrombo-embolic occlusions of cerebral blood vessels and the activation of a complex cascade of events resulting in a permanent brain damage [7, 8, 11–15].

**Fig. 1:**
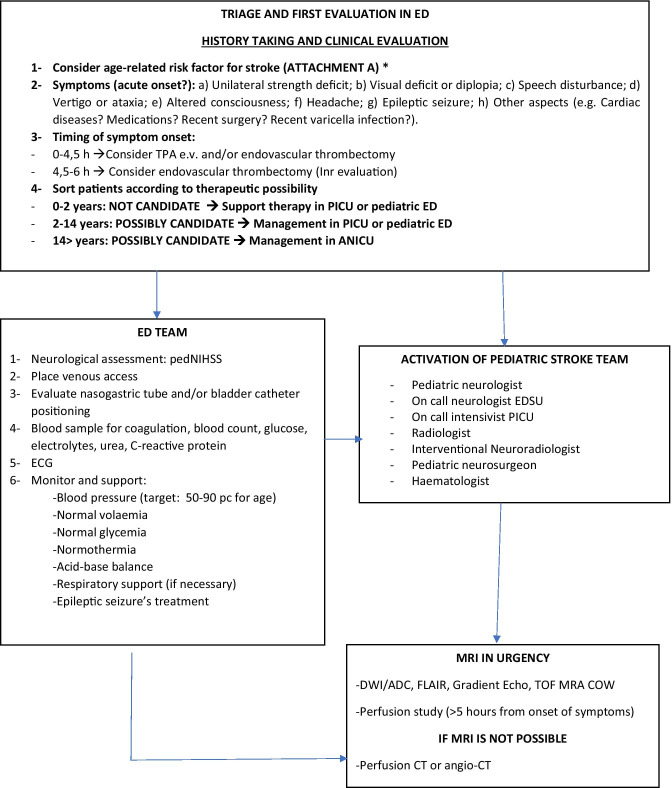

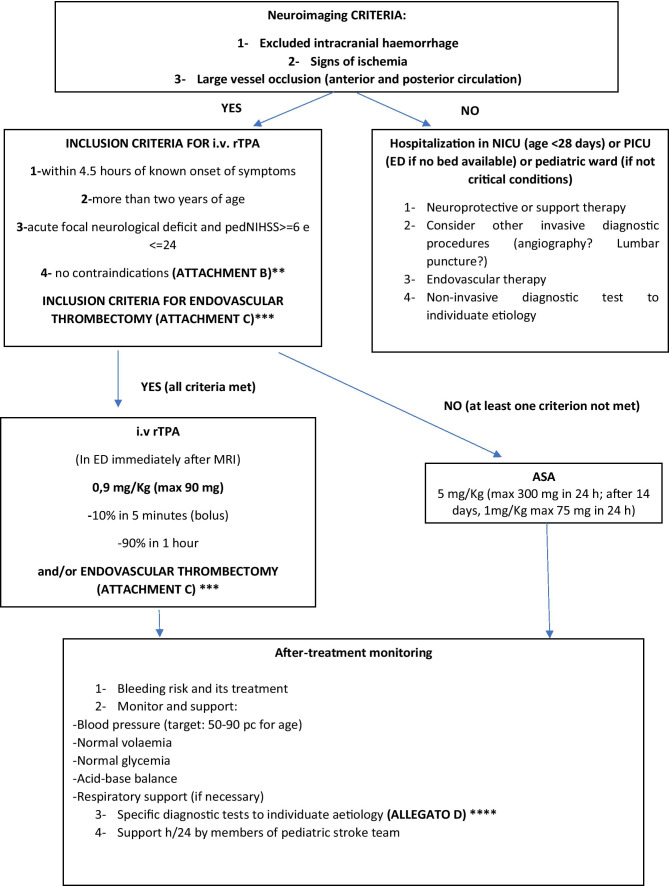

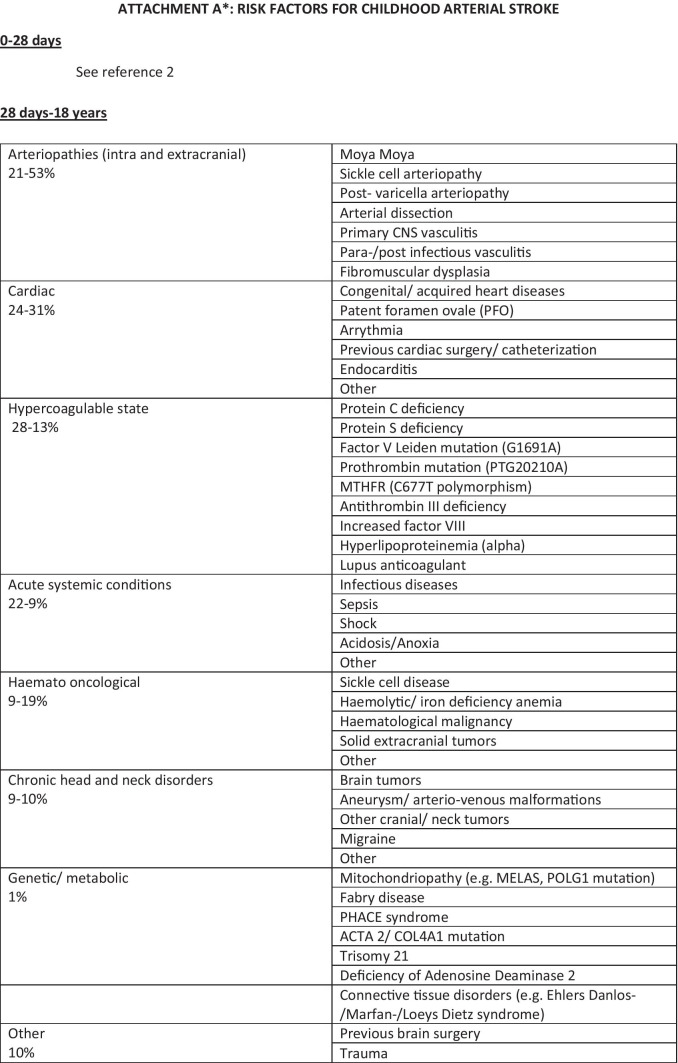

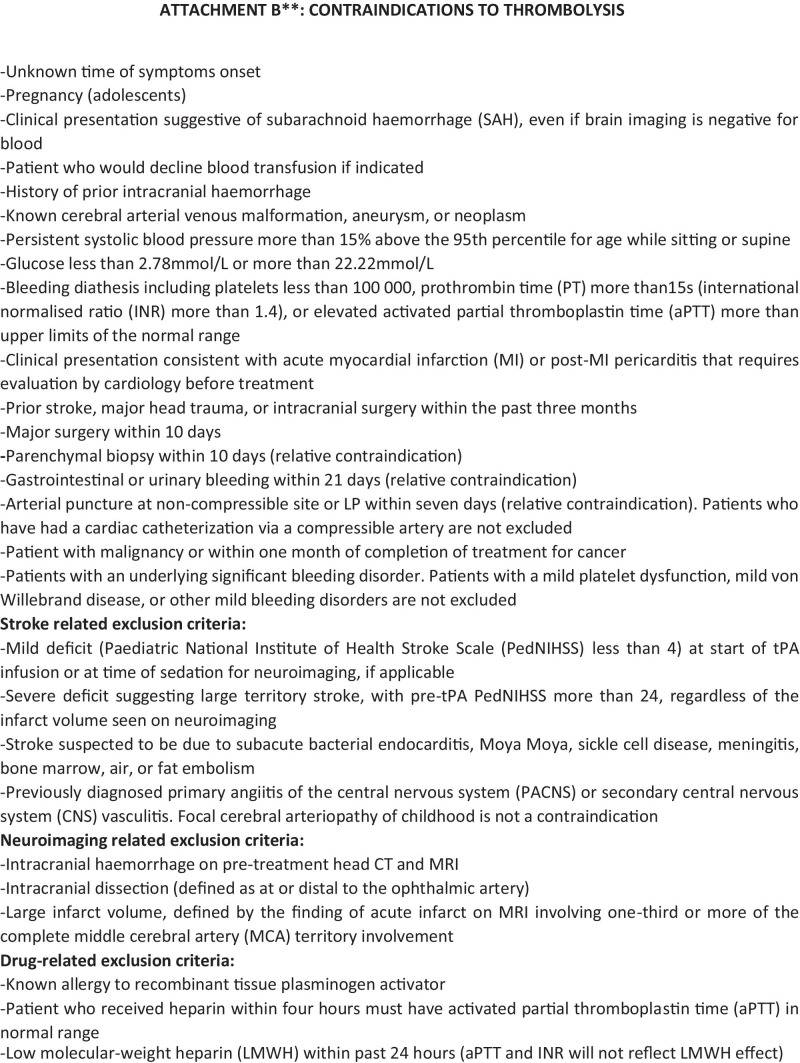

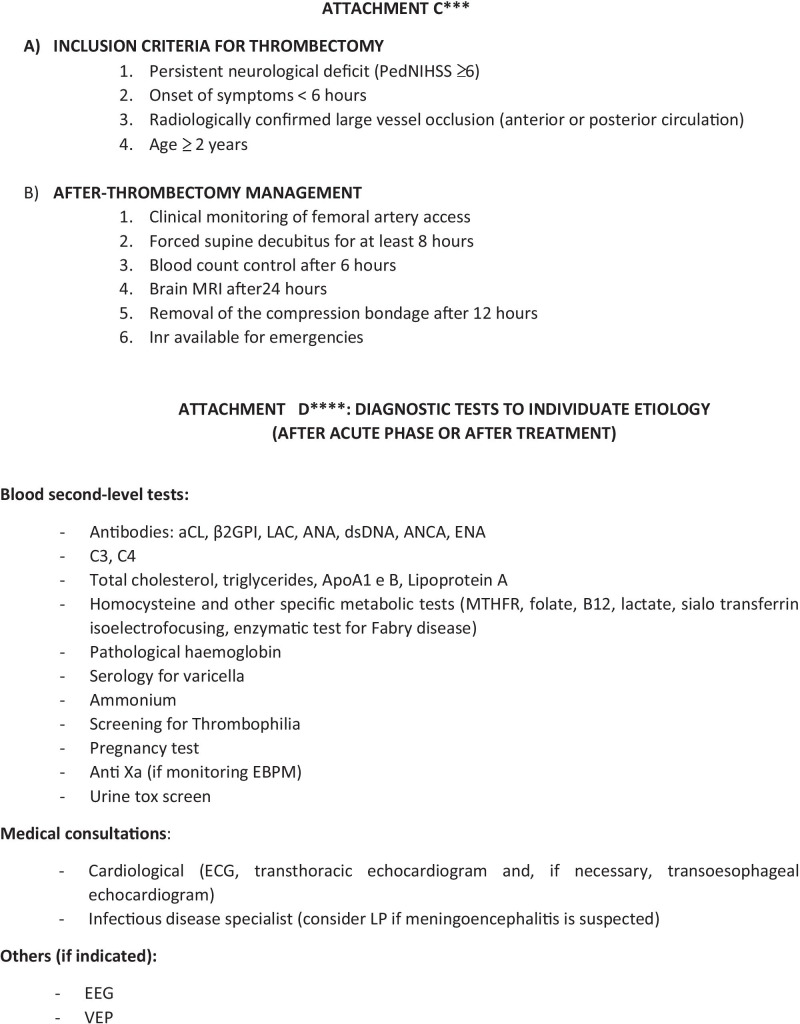
A suggested protocol for the diagnostic work-up and the treatment of acute ischemic stroke in children. Legend: ED Emergency Department, rTPA recombinant tissue plasminogen activator, PICU pediatric intensive care unit, ANICU adults neurological intensive care unit, NICU neonatal intensive care unit, pedNIHSS Pediatric National Institute of Health Stroke Scale, ECG electrocardiogram, PCR protein C reactive, Inr interventional neuroradiologist, MRI magnetic resonance imaging, DWI diffusion weighted imaging, ADC apparent diffusion coefficient, FLAIR fluid attenuated inversion recovery, TOF time of flight angiography, MRA magnetic resonance angiography, COW circle of Willis, CT computed tomography, ASA acetylsalicylic acid, PT prothrombin time, INR international normalized ratio, aPTT activated partial thromboplastine time, LP lumbar puncture, CNS central nervous system, MCA middle cerebral artery, LMWH low molecular-weight heparin

The main risk factors in children older than 28 days and their respective frequencies are summarized in Fig. 1—Attachment A. Non atherosclerotic arteriopathies, cardiac disorders, and prothrombotic states account for most of the cases, with a variable distribution of their frequency in different geographical areas or age-ranges [14].

Data collected from the International Pediatric Stroke Study, a worldwide prospective study on 676 pediatric patients between 0 and 18 years, evidenced a higher prevalence of acute systemic conditions (including dehydration, sepsis, fever, acidosis, shock, anoxia/asphyxia, viral gastroenteritis) in Asia and South America and a lower prevalence of arteriopathies in Asia and chronic systemic conditions (haematological, oncological, and genetic disorders) in Europe and Australia [15]. Non atherosclerotic arteriopathies were the predominant aetiology in all the age ranges, with the highest prevalence in children between 5 and 9 years old, while the highest prevalence of cardiac disorders and acute conditions were reported in patients under the age of 5 [14, 16]. The term “non atherosclerotic arteriopathies” included a group of heterogeneous disorders resulting in lesions or structural abnormalities involving the cerebral blood vessels’ wall as a consequence of infectious, parainfectious or inflammatory mechanisms but also genetic predisposition or vascular malformation (e.g., focal cerebral arteriopathy, PHACE, sickle cell disease, post-varicella arteriopathy, fibromuscular dysplasia) [17]. The VIPS (Vascular Effect of Infection In Pediatric Stroke Study) study identified viral infections in the prior week, recent vaccination, black ethnicity, and rural residence as risk factors for a higher occurrence of arterial ischemic stroke in children [18, 19]. Serological evidence of recent, and mostly asymptomatic, herpesvirus infections were detected in 45% of the enrolled patients with a predominance of HSV1 and HSV2 over VZV (respectively 24.5% versus 11.3% of the cases) [20].

A large prospective cohort study by DeVeber et al. that recruited 894 children with stroke in Germany, Canada, and UK, aiming to determine the association between prothrombotic conditions and risk of recurrent episodes of stroke, evidenced a recurrence rate of 17.9% between 1 day and 136 months after the first stroke [21]. The following conditions were identified as independent risk factors for recurrence: antithrombin deficiency (hazard ratio 3.9; 95% confidence interval 1.4–10.9), increased Lipoprotein(a) (hazard ratio 2.3; 95% confidence interval 1.3–4.1) and more than one prothrombotic marker (hazard ratio 1.9; 95% confidence interval 1.1–3.2) [21].

Cardiac conditions associated with stroke encompass a wide spectrum of either acquired or congenital diseases. A study by Rodan et al., that recruited 135 patients with congenital heart diseases and a diagnosis of acute ischemic stroke from Canadian Pediatric Ischemic Stroke Registry, reported a 10-year-recurrence rate of 27% [22]. The most common associated risk factors included mechanical heart valve (hazard ratio = 8.8), systemic infection (hazard ratio = 5.7), and prothrombotic-state (hazard ratio = 2.9) [22]. Asaki et al. identified two additional risk factors in a retrospective case–control study on 52 cardiopathic children who developed an arterial ischemic stroke after invasive procedures: length of ICU hospitalization and post-procedural infections [23].

## Clinical presentation

Childhood stroke may present, similarly to adults, with several localizing signs and symptom (hemiparesis or hemifacial weakness, speech or language dysfunctions, vision disturbances, or ataxia) even if non localizing manifestations and seizures have a higher frequency in pediatric age, especially in children under the age of 6. (Table 1) [2, 8, 16]. Focal signs (numbness or weakness) are the most common clinical presentations at the onset in all the age ranges [5, 8]. Diffuse signs have a more variable distribution in the different age ranges: seizures appear in more than half of infants under the age of 12 months (56–75%), whereas headache is a frequent complaint in school-age children (33–50%) [5, 8]. In the first months of life, seizures (mainly brief focal episodes) and tone abnormalities (either increased or decreased muscle tone) are frequent clinical hallmarks, while focal symptoms and signs of encephalopathy may occur in a lower proportion of the cases and silent episodes without relevant symptoms are also possible [7, 11, 24].

**Table 1:** Frequency of signs and symptoms in children with arterial ischemic stroke ^**2,8,16**^

Frequency of signs and symptoms in children with arterial ischemic stroke (%)	
Focal signs/symptoms 82–85%	Hemiparesis 72%
	Facial weakness 41%
	Speech disturbances 20–50%
	Visual disturbances 5–15%
	Ataxia 8–10%
	Other 19%
Non-localizing features 61–64%	Altered mental status 17–42%
	Headache 23–50%
	Vomiting 10%
	Papilledema 1%
	Other 8%
Seizures 15–31%	Focal 20%
	Generalized 11%
	Both focal and generalized 2%

The most used clinical adult stroke assessment tools were not extensively validated in pediatric patients, and their predictive value remains uncertain [13]. Moreover, these tools are insensitive of posterior circulation defects as they do not assess ataxia, dysarthria, and visual field disturbances [13]. Pediatric National Institute of Health Stroke Scale (PedNIHSS) is the standard tool for the emergency evaluation of stroke severity [3]. It is a score-based system exploring 11 neurological domains (level of consciousness, best gaze, visual function, signs of facial palsy, motor function of arms and legs, limb ataxia, sensory functions, language, dysarthria, and extinction/inattention) that was associated with an excellent interrater reliability in a multicenter prospective cohort performed by trained child neurologists in 2011[13]. Mackay and colleagues evaluated the applicability of ROSIER (Recognition of Stroke in the Emergency Room) and FAST (Face Arm Speech Test) scales in a cohort of radiologically confirmed children and found a sensitivity for stroke detection of 81% and 76%, respectively [25]. The same group reported poor predictive values for ROSIER and Cincinnati Prehospital Stroke Scale (CPSS) in discriminating between stroke and mimics in children presenting with acute neurological symptoms [26].

In a prospective observational study of 287 children presenting to the emergency department with acute onset focal neurologic symptom, Mackay et al. observed that stroke accounted for 7% of the aetiologies (versus 28% for migraine, 15% for seizures, 10% for Bell palsy, and 6% of conversion disorders) [27]. In a subsequent study, the same group compared 102 children with ischemic stroke/TIA and 280 children with other neurological symptoms mimicking acute stroke. The clinical predictors that were significantly associated with stroke included normal clinical status in the week before presentation (OR 5.76, 95% CI 2.25–14.79), arm and face weakness (OR 8.66, 95% CI 2.50–30.02 and OR 2.94, 95% CI 1.19–7.28, respectively), and inability to walk (OR 3.38, 95% CI 1.54–7.42) [28]. Seizures and loss of consciousness were not independently associated either with stroke or with mimics, suggesting that these symptoms might not be used as discriminator for diagnosis [28].

## Neuroimaging

**Fig. 2:**
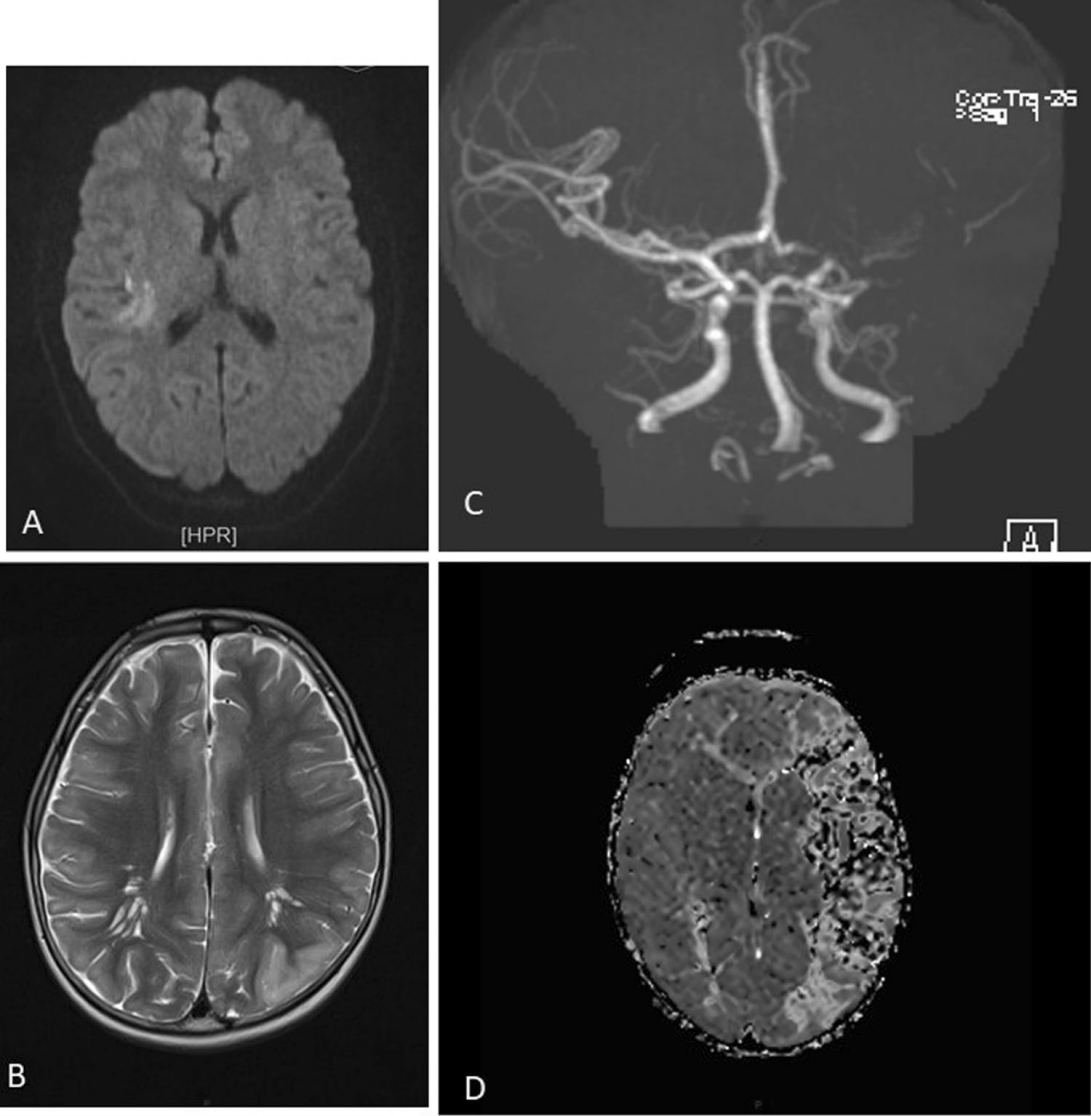
**A** Right parietal FLAIR-hyperintense lesion in a 14-year-old girl who presented with a complete anaesthesia of the left forearm and hand, visual impairment and headache. **B** Left temporo-occipital T2-lesion in a 6-year-old boy who presented with aphasia. **C** and **D** Angio-MRI and PWI sequences in a 2-year-old boy with the occlusion of M1 tract of the left-middle cerebral artery and of the A1 tract of the ipsilateral anterior cerebral artery

The acquisition of urgent MRI images is always necessary to achieve a rapid differential diagnosis (in children stroke-mimics have a higher frequency than in adults) and to decide the more suitable acute therapy (after having identified the areas of ischemia, penumbra, and/or large vessel occlusion and after having excluded intracranial haemorrhages) (Fig. 1) [2]. Current guidelines state that brain MRI should always be preferred to CT because this last one has a poor sensitivity with a missed diagnosis in 44–83% of cases [2, 27, 29, 30]. Figure 2 shows some illustrative MRIs that were performed in our Institution.

“Hyperacute MRI protocols,” including diffusion weighted imaging (DWI) and apparent diffusion coefficient maps (ADC) sequences, were developed also for children and demonstrated a good potential in detecting a reduced diffusivity in the involved arterial territories [31]. Perfusion MRI techniques, such as arterial spin labelling (ASL) sequences, can be appealing for children because allowing to measure relative cerebral blood flow and volume and detect areas of ischemia without the use of a contrast agent [32]. ASL has been demonstrated to correlate with the degree of stenosis, infarct location, diffusion restriction, and follow-up T2 infarct volumes [32].

The distribution of the lesions might help physicians to identify several common aetiologies occurring in the pediatric age [33]. For instance, strokes in children with cardiac disorders are often bilateral, involving both anterior and posterior circulation, with an increased tendency to a hemorrhagic transformation [33]. Herpesviruses-related strokes are generally multifocal, with an involvement of the limbic system and basal ganglia associated with leptomeningeal enhancement, reflecting meningitis [33]. The sickle cell disease (SCD)-related strokes are generally located at the border-zone between the ACA and MCA territories, with relative sparing of the posterior circulation [34].

## Treatment

Randomized clinical trial assessing safety and efficacy of thrombolysis and/or endovascular treatment were never performed for pediatric age [35, 36].

The most updated pediatric stroke guidelines state that it is feasible to apply rTPA in children from 2 years of age, with persistent disabling neurological deficits (e.g., Pediatric NIH Stroke Scale score ≥ 6 at the time of intervention) and radiographically confirmed cerebral large artery occlusion, within 4.5 h of known onset of symptoms [2, 35, 36]. The suggested drug dosage is the same used for adults, although according to known age-related differences in the fibrinolytic system with children having higher levels of tissue plasminogen inhibitor than adults, it would be reasonable to consider that children might benefit of higher dosages of rTPA [2, 13].

The Thrombolysis in Pediatric Stroke (TIPS) trial was designed as a 5-year prospective multicentre trial to study the safety and pharmacokinetic profile of intravenous rTPA in pediatric patients with acute ischemic stroke [37]. Although it was closed prematurely due to lack of enrolment, it favoured the formation of paediatric stroke centres and acute paediatric stroke protocols [37]. A 2020 study by Amlie-Lefond et al. retrospectively collected data on children treated with intravenous rTPA for arterial ischemic stroke in 16 former TIPS sites [38]. It showed that the overall risk of symptomatic intracerebral haemorrhage after intravenous rTPA, when given within 4.5 h after symptom onset, was as low as 2.1% (compared with 6.4% reported for adults) [38, 39]. Symptomatic intracerebral haemorrhages were defined if ECASS (European Cooperative Acute Stroke Study) II parenchymal hematoma type 2 or any intracranial haemorrhage associated with neurological deterioration within 36 were observed after tPA administration [39].

Several differences in adult versus childhood stroke might influence the performance of endovascular thrombectomy (i.e., large vessel occlusion, collateral vessel status, intracranial vessel sizes in children) [2, 40]. The 2020 Save ChildS study provided multicentre evidence for the use of mechanical thrombectomy in children with large vessel occlusion ischemic stroke [39]. This study was focused on the potential effect modification of different thrombectomy techniques, (A Direct Aspiration First Pass Technique [ADAPT] and non-ADAPT groups) as well as different device sizes, suggesting that the global neurological outcome was generally positive regardless of any specific device and approach selection [41]. No remarkable differences were found between ADAPT and non-ADAPT group in terms of rate of successful recanalization (85.7% versus 87.5%), PedNIHSS on day 7, and modified Rankin scale at the discharge and after 6 and 24 months [41].

No new insights were developed in the last decades about standard of cares when thrombolysis, or endovascular treatments are not feasible with most of the suggested protocols being based on traditional anti-thrombotic therapies (mainly low-molecular weight heparins and acetylsalicylic acid) [2, 35, 36]. The lacking initiation of an anti-thrombotic treatment for stroke prevention is associated with a 1.5–2.5 fold increased risk of recurrences after a first episode [2]. Anti-thrombotic therapies are contraindicated in the acute setting when haemorrhagic stroke has not been excluded and in other cases including Moya Moya disease, surgery within the previous 24 h, methotrexate toxicity, thrombocytopenia with platelet count less than 50,000/mm^3^, or history of heparin-induced thrombocytopenia. [2].

The long-term treatment is commonly based on low-molecular weight heparins or acetylsalicylic acid without clear data suggesting which one of the two therapies is better in children and safety data supporting both approaches [2]. A longer-term prevention therapy should be considered in patients with genetically determined thrombophilia (e.g., hyperhomocysteinemia, protein C deficiency, antithrombin deficiency) or in children with a stroke following congenital heart diseases, while in the other cases, the duration of the treatment may depend on the underlying aetiology with a usual preferred temporal range between 2 years and a lifetime [2]. No data about the prophylactic use of direct oral anticoagulants (e.g., antithrombin agent or anti–factor Xa agents) in the paediatric age were collected [2].

Specific prevention therapies may be considered for selected aetiologies (e.g., transfusions or hydroxyurea for sickle cell disease, L-arginine for MELAS, agalsidase or migalastat for Fabry disease, pyridoxine in combination with folic acid and vitamin B12, methionine-restricted, cystine-supplemented diet and betaine, for homocystinuria) even if adequate supporting evidences are often not available because of their rare occurrence [2, 42]. The usefulness of other specific strategies (e.g., anticoagulation for pediatric arterial dissection, patent foramen ovale closure, steroids for focal cerebral arteriopathies, or surgical strategies for moyamoya) remain unsolved controversial [2].

## Management protocols

A suggested protocol for the early evaluation, treatment, and diagnostic work-up of children with ischemic arterial stroke, that was developed in our institution, is illustrated in Fig. 1.

The activation and implementation of paediatric acute stroke protocols speed up stroke recognition and diagnosis in children, making the delivery of hyperacute recanalization treatment feasible [43–49]. A recent paper by Rivkin et al. retrospectively compared children presenting with acute neurological symptoms to their tertiary children’s hospital before and after the implementation of their stroke alert system [45]. The median time from emergency department arrival to neuroimaging was significantly lower for patients evaluated after the implementation of the stroke pathway (82 min; IQR, 54–123 min after [*n* = 65] vs 196 min; IQR, 85–230 mi before [*n* = 14]; *P* < 0.01), thus enabling a greater proportion of them to lay within the time frame for acute intervention [43]. Similar results have been published by Tabone et al. with a reported mean time of 165 min from symptoms onset to MRI for 13 consecutive children who underwent acute recanalization treatments (i.v. rTPA, endovascular procedure or both) as part of a regional pediatric acute stroke protocol in France [44].

The most recent protocols have all been similarly developed from multidisciplinary working groups involving paediatric and adult specialists (emergency physicians, stroke unit fellows, neurologists, interventional and diagnostic neuroradiologists, anaesthesiologists) [43–46]. All the protocols were organized in three diagnostic steps based on (a) the quick assessment of the patient at the point of care (either emergency departments, intensive care units, or in-hospital wards), (b) the transfer to the MRI suite where pre-determined hyperacute MRI stroke protocols are being performed, and (c) the determination of eligibility for recanalization therapy, according to the presence/absence of inclusion/exclusion criteria (see Attachment C in Fig. 1) [51–52]. Table 2 summarizes the protocols that were published in the last 5 years [43, 44, 46–49]. The application of all these protocols resulted in a neuroradiologically confirmed diagnosis in almost all the patients within the correct time window for the application of acute recanalization therapies [43, 44, 46–49]. Despite these data, relevant differences in the treatment of choice emerged among the involved institutions, where the expertise for thrombolysis/thrombectomy was not always available [43–49]. None of the studies provided adequate information about the proportion of successfully treated patients, the frequency of treatment-related complications, and long-term outcome [43–49].

**Table 2:** Main published institutional protocols about the management of pediatric stroke in the last 5 years ^**49, 50, 52, 53, 54, 55**^

Article	No. of enrolled patients	Number of patients diagnosticated arterial ischemic stroke	Time of application of protocol	Main symptoms at onset	PedNHISS	Time to neuroimaging	Number of treated patients	% of success of treatment	Number of patients with complications
Rivkin et al. (2019)^49^	65 pt presenting with brain attack symptoms	6	2.5 years	Focal motor symptoms, sensory deficit, headache	< 4 (65% patients); > 4 (32% patients); not documented in 3 patients	82 min	Not available	Not available	Not available
Tabone et al. (2017)^50^	13 patients with acute ischemic stroke	13	3.3 years	Focal motor symptoms, language disorder	Median pedNIHSS 10	165 min	15 (11 pt rTPA, 4 pt endovascular procedure)	Not available	No intracranial or peripheral bleeding after treatment. One early death (malignant stroke). mRS score at 3 months 0–2
Ladner et al. (2015)^55^	124 stroke alerts	21	3 years	Focal motor symptoms, altered mental status, headache	Not available	94 min (to MRI) 59 min (to CT)	11 (1 pt rTPA, 2 pt mechanical thrombectomy)	Not available	Median pediatric stroke outcome measure 0.75 (IQR, 0–2.13), mild-to-moderate ongoing neurological deficits with effect on function
DeLaroche et al. (2016)^52^	36 stroke alert activations	7	4 years	Neurological deficit, headache, altered mental status, gait abnormalities, seizures	Not available	46 min (to CT) 320 min (to MRI)	Not available	100%	None patient reported complications (64% of pts are discharged home. 36% to in-patients rehabilitation. No deaths)
Shack et al. (2016)^53^	112	122	7 years	Not available	Median pedNIHSS 7 (4–12)	90 min	Not available	Not available	Not available
Wharton et al. (2020)^54^	385 stroke alert activation	80 stroke (not specified acute ischemic/haemorrhagic)	7 years	Not available	Median pedNIHSS 7.5–10	79 min	57 (0 pt rTPA, 4 pt endovascular procedure)	Not available	Not available

## Outcome

Recent studies reported a mortality rate after childhood stroke between 2.6 and 5% [4, 50]. The large Canadian prospective stroke registry documented the emergence of persistent neurological deficits in 60% of neonates and 70% of children, of which, 36% were mild, 23% were moderate, and 10% were severe deficits [4]. In older children, the observed deficits remained mostly stable (with a reduction in their deficit severity in 16% and an increase in 8% of them) and recurrent arterial ischemic stroke or TIA were reported in 12% of the cases [4]. In another large population-based cohort study, Fullerton et al. observed that although recurrences were rare after perinatal stroke, one-fifth of arterial ischemic stroke recurred in later childhood (with a 5-year cumulative recurrence rates of 1.2% after perinatal stroke and 19% after later childhood stroke) [51].

A recent review of 22 studies on newborns and children with stroke evidenced lower executive functions performances than typically developing patients [52]. The long-term evolution of executive functions impairment was scarcely explored (only two longitudinal studies with small cohorts of patients), and no concordant evidence were collected about a possible impact of factors such as age at stroke onset, time since stroke, and lesion characteristics [52]. Inhibition resulted as more impaired than working memory processes or cognitive flexibility, independently by the age at stroke onset or lesion features [52].

## Conclusions

Physicians approaching to ischemic pediatric stroke should consider several differences versus adults that represent remarkable clues for the diagnosis and the treatment: (1) the more significant etiopathogenic role of intracranial non-atherosclerotic arteriopathies, thromboembolic complications of congenital cardiopathies, and haematological disorders such as sickle cell’s disease or coagulopathies; (2) the high frequency of atypical presentations in children (e.g., headache only or no evident clinical symptoms); and (3) the lack of validated guidelines for thrombolytic and endovascular treatments in paediatric age with obvious negative consequences on the prevention of permanent neurological sequelae.

## Abbreviations

MRI: Magnetic resonance imaging
VIPS: Vascular Effect of Infection In Pediatric Stroke Study
PHACE: Posterior fossa brain malformations, Hemangioma, Arterial lesions, Cardiac abnormalities, and Eye abnormalities
HSV1: Herpes simplex virus 1
HSV2: Herpes simplex virus 2
VZV: Varicella-Zoster virus
TIA: Transient ischemic attack
OR: Odds ratio
CI: Confidence interval
PedNIHSS: Pediatric National Institute of Health Stroke Scale
ROSIER: Recognition of Stroke in the Emergency Room
FAST: Face Arm Speech Test
CPSS: Cincinnati Prehospital Stroke Scale
CT: Computed tomography
DWI: Diffusion weighted imaging
ADC: Apparent diffusion coefficient
ASL: Arterial spin labelling
SCD: Sickle cell disease
ACA: Anterior cerebral artery
MCA: Middle cerebral artery
rTPA: Recombinant tissue plasminogen activator
TIPS: Thrombolysis in Pediatric Stroke
ECASS: European Cooperative Acute Stroke Study
ADAPT: A Direct Aspiration First Pass Technique
IQR: Interquartile range
